# Case report: Combinations of immune checkpoint inhibitor, chemotherapy, and hyperthermia therapy avoid lymphatic recurrence in cholangiocarcinoma

**DOI:** 10.3389/fonc.2024.1421340

**Published:** 2024-10-24

**Authors:** Heng-Jui Chang, Chiao-Hsu Ke, Yu-Shan Wang

**Affiliations:** ^1^ Department of Radiation Oncology, Wesing Surgery Hospital, Kaohsiung, Taiwan; ^2^ Department of Chemical Engineering and Biotechnology, Institute of Chemical Engineering, National Taipei University of Technology, Taipei, Taiwan; ^3^ Department of Veterinary Medicine, School of Veterinary Medicine, National Taiwan University, Taipei, Taiwan; ^4^ Uni-Pharma Co-Ltd., Taipei, Taiwan

**Keywords:** immune checkpoint inhibitor, hyperthermia, modulated electro-hyperthermia, cholangiocarcinoma, immunotherapy

## Abstract

Cholangiocarcinoma is a malignancy known for its aggressiveness and limited treatment options. The malignant tumor behaviors include intrahepatic recurrence, regional lymph node (LN) metastasis, peritoneal carcinomatosis, and lung metastasis. Herein, we reported a case of lymphatic recurrence in an intrahepatic cholangiocarcinoma patient after surgery, adjuvant concurrent chemoradiotherapy (CCRT), who experienced a remarkable response to a combination therapy. However, the patient failed to undergo radiotherapy or other invasive local therapy and therefore received Opdivo (nivolumab) in combination with chemotherapy (FOLFOX) and modulated electro-hyperthermia. Notably, after these medical interventions, this patient had a complete response (CR) to treatments, in which no lymph node metastasis occurred, and a significantly decreased tumor marker, CA 19-9, level was found. This case highlights the potential of multiple anti-tumor therapies, including immune checkpoint inhibitors, chemotherapy, and hyperthermia, in managing challenging cholangiocarcinoma cases.

## Introduction

Cholangiocarcinoma is a formidable type of cancer that arises in the bile ducts and is known for its rarity and aggressiveness. However, patients with cholangiocarcinoma are often diagnosed in an advanced stage, which restricts treatment options and influences the overall survival time. Following curative resection, the median survival time is reported to be 28–30 months, with a roughly 30% 5–year survival rate (1). The 5–year survival rate of intrahepatic cholangiocarcinoma with regional lymph node (LN) metastasis is about 9%, and only 3% for distant metastasis (2). One of the most significant challenges in managing this cancer is treating lymphatic recurrence following surgery and postoperative radiotherapy. Fortunately, a recent case report provides evidence that combining immunotherapy, chemotherapy, and modulated electro-hyperthermia may be an effective strategy for dealing with local lymphatic recurrence in cholangiocarcinoma (3).

Conventional treatments for cholangiocarcinoma, such as chemotherapy with gemcitabine and cisplatin, typically result in modest response rates and limited progression-free survival (PFS). For unresectable cholangiocarcinoma, the median overall survival (OS) with this regimen is less than one year, and the 5-year survival rate ranges from 20% to 35% depending on various factors like tumor location and stage (4). Response rates to systemic chemotherapy alone are generally low, with PFS often being less than six months. Studies have shown that adjuvant chemotherapy, despite its use, has not consistently demonstrated significant improvements in overall survival for all patients. The variability in outcomes is influenced by factors such as surgical margins, lymph node involvement, and the presence of metastatic disease (5). Locoregional therapies, including ablation, radioembolization, and chemoembolization, offer alternative strategies, particularly for patients who are not candidates for surgery. These therapies can provide local control and symptom relief, and in some cases, they achieve comparable outcomes to surgical resection for early-stage disease (6). The integration of newer treatment modalities, such as immunotherapy and targeted therapy, is being explored to improve response rates and survival outcomes in cholangiocarcinoma. However, the effectiveness of these treatments varies, and more research is needed to establish their role in standard care (4).

## Case presentation

A 62-years-old male had history of hypertension under regular medication control. There were no other underlying medical comorbidities that this patient was suffering from diabetes, renal disease, cerebrovascular disease, hepatitis B/C carriers, congestive heart failure, or any other neoplastic diseases. His lifestyle factors included a regular, balanced diet, moderate stress level, no alcohol or tobacco use, no insomnia, and moderate physical activity. He initially presented with mild abdominal dull pain, and a computed tomography (CT) scan revealed a large intrahepatic tumor measuring 7–8 cm in size. It might have involved LNs in February 2021. At that time, tumor marker CA 19-9 exceeded 3000 U/mL (ranging from 0–37 U/mL in normal individuals). He underwent hepatectomy along with LN dissection in March 2021, followed by postoperative concurrent chemoradiotherapy (CCRT). The pathology report demonstrated a 7 cm cholangiocarcinoma with 1 in 9 regional LN metastasis, pT2N1, stage IIIb (AJCC 8^th^ edition). Until July 2021, adjuvant CCRT was carried out using a 4500cGy/25fx radiation dose to the tumor bed, lymphatic region, and gemcitabine 800-1000 mg/m^2^ for seven cycles. CA 19-9 reached a nadir of 27 U/mL then. However, less than three months after the initial treatment, the patient experienced a single regional LN recurrence, measuring a maximum of 2.76 cm. It was situated between the pancreas, duodenum, superior mesentery artery, inferior vena cava, and abdominal aorta. CA 19-9 rapidly increased to 195 U/mL in October 2021. Because of the prior radiation exposure, there was no room for further radiotherapy. It was also impractical to use any other invasive local therapies.

## Treatment approach

**Figure 1:**
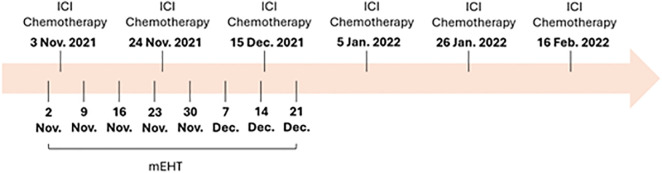
The treatment timeline of this case. ICI, Immune checkpoint inhibitor; mEHT, modulated electro-hyperthermia.

Because of the patient’s limited treatment options, a multidisciplinary team recommended a combination therapy to target the recurrent lymphatic metastasis. Immunotherapy with nivolumab, a PD-1 inhibitor, and chemotherapy with the FOLFOX regimen were chosen based on their known benefits in treating cholangiocarcinoma. Additionally, modulated electro-hyperthermia (mEHT) was employed to enhance the effectiveness of these treatments by selectively targeting cancer cells and making them more susceptible to therapy. The treatment protocol was described as follows (**Figure 1**).

Immune checkpoint inhibitor: The patient was initiated on nivolumab (Opdivo), a programmed cell death protein-1 (PD-1) immune checkpoint inhibitor. Nivolumab was administered 100 mg intravenously every three weeks for six cycles until February 2022. This immunotherapy aims to activate the patient’s immune system to recognize and attack cancer cells.

Chemotherapy: The patient underwent intravenously six cycles of chemotherapy (FOLFOX) every three weeks until February 2022, consisting of a platinum-based regimen tailored to cholangiocarcinoma. The FOLFOX regimen included oxaliplatin 85 mg/m^2^, leucovorin 400 mg/m^2^, and fluorouracil (5-FU) 2400 mg/m^2^ bolus, followed by 5-FU 600 mg/m^2^ as a 46-hour continuous infusion. Chemotherapy aimed to further debulk the tumor and sensitize it to immune checkpoint inhibition.

Modulated electro-hyperthermia (mEHT): The patient underwent a total of eight sessions of modulated electro-hyperthermia between November 2, 2021, and December 21, 2021, a hyperthermia treatment that applies controlled electromagnetic fields to target cancer cells selectively. The duration of each mEHT session lasted for 60 minutes. The EHY-2000 model of the Oncotherm machine was used for the mEHT treatments. The machine operated at a frequency of 13.56 MHz. The power output was maintained below 150 watts to ensure selective targeting of cancer cells while sparing healthy tissues. The mEHT treatments were administered specifically to the site of lymphatic recurrence. This approach leverages controlled electromagnetic fields to increase the temperature of cancer cells selectively, causing stress on their cell membranes and leading to cell death. This selective heating enhances the effectiveness of concurrent treatments, such as immunotherapy and chemotherapy, by making the cancer cells more susceptible to these therapies.

## Outcome

The combined treatment approach yielded remarkable results, including a complete response to treatments and a decreased tumor marker.

**Figure 2:**
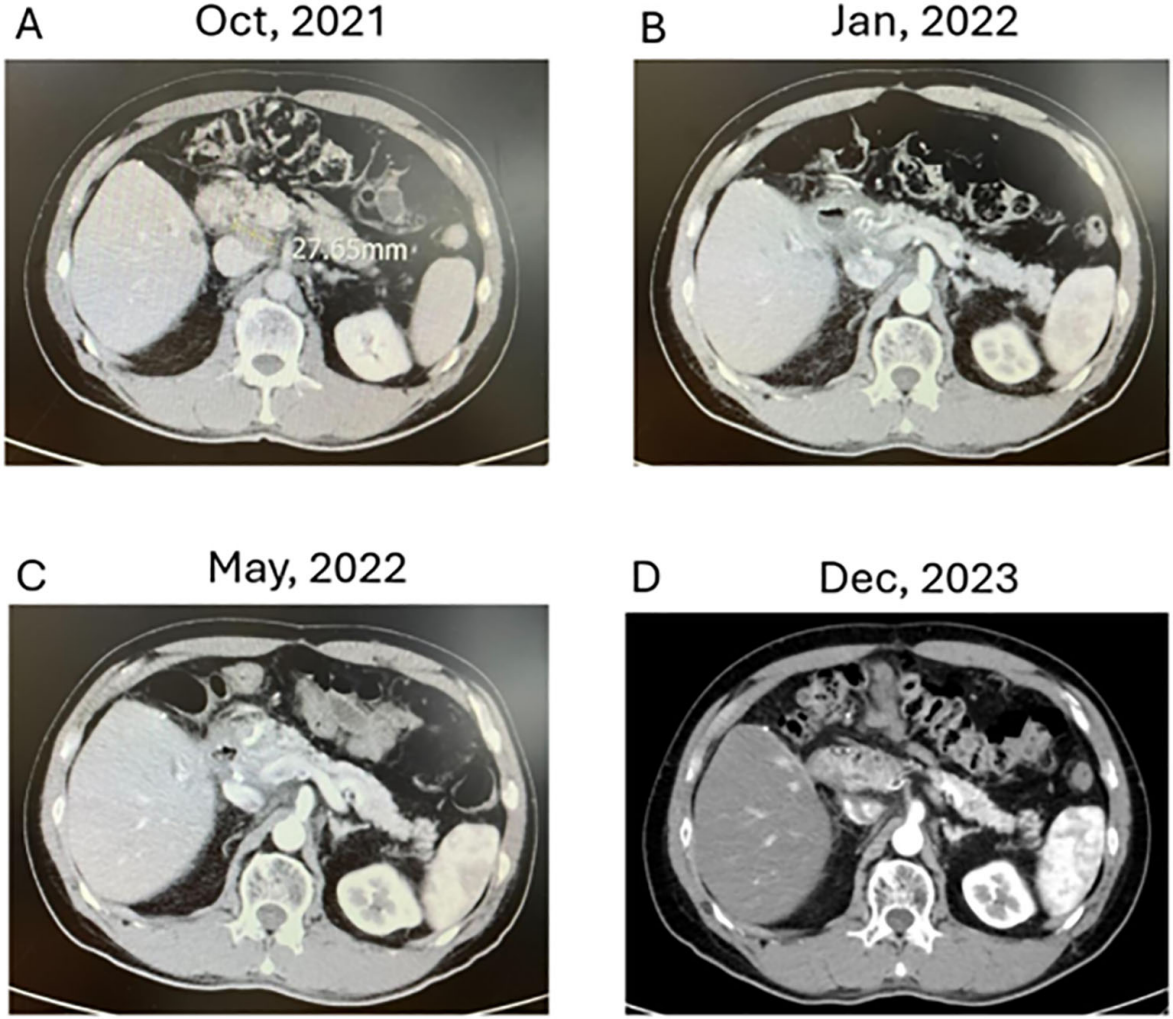
Evolution of Cholangiocarcinoma Through Multimodal Therapy **(A–D)** depict axial CT images demonstrating the response of lymphatic recurrence in a cholangiocarcinoma patient over time. (**A**: October 2021): Displays a single lymph node recurrence measuring approximately 2.76 cm amid critical anatomical structures post-adjuvant CCRT. (**B**: January 2022): Reveals a marked decrease in lymph node size, nearing complete response, following the initiation of combined modality treatment, including immune checkpoint inhibitors, chemotherapy, and modulated electro-hyperthermia. (**D**: December 2023): Illustrates sustained therapeutic success with no detectable signs of recurrence upon seven months of follow-up. (**D**: December 2023): At 26 months post-recurrence, comprehensive imaging confirms the absence of lymphatic or other metastases, supporting the patient’s status in clinical remission.

Complete response: Imaging studies revealed complete regression of LN metastasis in response to the combined therapy regimen. The lymphatic recurrence was no longer detectable. Twenty-six months after recurrence, a CT scan in December 2023 still demonstrated no evidence of disease (**Figure 2**).

**Figure 3:**
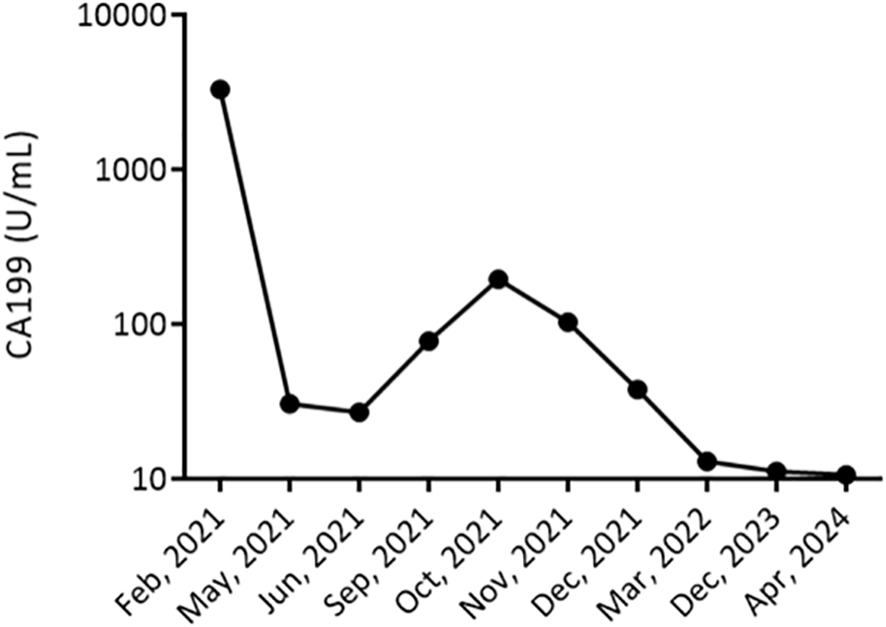
Longitudinal Monitoring of Tumor Marker CA 19-9 Levels. This figure displays the CA 19-9 tumor marker levels trend in a patient with cholangiocarcinoma from February 2021 to December 2023. The graph represents a significant decrease in CA 19-9 levels, from >3000 U/mL in February 2021, indicating a high tumor burden, to stabilizing at 13 U/mL by December 2023. The marked reduction in CA 19-9 levels post-treatment suggests an effective response to the therapeutic regimen.

Tumor Marker Response: Tumor marker CA 19-9, which had initially elevated at 195 U/mL, exhibited a significant decrease, reaching 12.9 U/mL after completing the treatment regimen. In the long-term follow-up, CA 19-9 reached a nadir of 11.2 U/mL in December 2023. As of April 2024, there has been no recurrence with a stable CA 19-9 value of 10.6 U/mL (**Figure 3**), and the disease-free period lasted 29 months.

## Discussion

Cholangiocarcinoma often presents a challenging clinical scenario when the cancer reoccurs, especially in the lymphatic system. In such cases, conventional treatments like radiation therapy and salvage surgery have limited efficacies, and alternative approaches should be taken into consideration This case study demonstrated how a multimodal approach that combines immune checkpoint inhibitors, chemotherapy, and mEHT therapy can achieve a favorable complete response and possibly cure the disease. The combined intervention approach targeted and activated the patient’s immune system to fight against cancer cells while minimizing the side effects that usually accompany such treatments. This novel approach offers a promising new avenue for managing cholangiocarcinoma recurrence in the lymphatic system.

More and more evidence has revealed the existence of oligo recurrence or oligometastatic, which is a transitional state between limited primary cancer and polymetastatic cancer. In this state, local therapy for metastatic lesions leads to a satisfactory survival rate on par with non-metastatic disease (7). Traditionally, radiotherapy, ablation therapy, and surgery are deemed to salvage local therapies for oligo-recurrent tumors. Recently, local electro-hyperthermia has also been introduced clinically as an alternative approach for the focal treatment of tumors, especially when radiotherapy is not feasible. Electro-hyperthermia could serve both curative and palliative purposes. Combining electro-hyperthermia with systemic therapy typically results in a higher response rate than systemic therapy alone. The mechanisms underlying electro-hyperthermia involve excitement and disruption of the cancer cell membrane, increasing drug infiltration and anti-cancer sensitivity, and creating massive cancer apoptosis (8). For advanced cholangiocarcinoma, immunotherapy has shown its efficacy when combined with chemotherapy, and this combination treatment approach has emerged as the new standard (9). The effectiveness of the immune response and cytotoxicity would be maximized if electro-hyperthermia could be added to this combined approach, and it may be possible to achieve a higher rate of complete response, curative status, and more prolonged survival for recurrent cholangiocarcinoma.

The benefits of hyperthermia in cancer treatment, particularly in enhancing immune responses and delaying drug resistance, have been well-documented in various studies. Hyperthermia, mainly used in the “fever range” (41-43°C), can influence cell membrane fluidity and stability and trigger a heat shock response. This response induces heat shock proteins (HSPs), which can protect tumor cells against apoptosis triggered by oxidative stress (10). Interestingly, hyperthermia at this temperature range can also promote apoptosis in tumor cells, balancing pro- and anti-apoptosis processes. This mode of cell death, known as immunogenic cell death (ICD), can induce an inflammatory response and enhance the immune system’s recognition of cancer cells (11). The process involves the release of damage-associated molecular patterns (DAMPs), such as high mobility group box 1 (HMGB1) and calreticulin (CRT), which are critical for the immunogenicity of cell death (12). The hyperthermia-induced elements are crucial because they are responsible for initiating tumor-specific immune responses, mainly by activating Toll-like receptor-4 (TLR-4) signaling pathways. HSPs, a group of highly conserved chaperone proteins, are among the most critical DAMPs induced by hyperthermia. These proteins, predominantly when overexpressed after hyperthermia, are associated with enhanced immune response (12). Furthermore, hyperthermia can suppress resistance pathways in cancer stem cells, making them more susceptible to other treatments like radiation therapy. This is particularly beneficial for bulky or refractory cancers. The cancer stem cell population plays a crucial role in disease recurrence due to its resistance to conventional therapies. By targeting these cells, hyperthermia can potentially improve treatment outcomes and reduce the likelihood of cancer recurrence (13).

Hyperthermia increases the expression of HSPs within tumor cells. HSPs play a crucial role in protein folding and protection under stress conditions, but they also enhance the immunogenicity of cancer cells (10, 14). When HSPs are overexpressed on the surface of tumor cells, they can improve antigen presentation, making the cancer cells more recognizable to the immune system. This heightened visibility enhances the effectiveness of immune checkpoint inhibitors like nivolumab, which relies on the immune system’s ability to identify and attack cancer cell (12). Hyperthermia can cause the release of tumor antigens due to the stress and subsequent apoptosis or necrosis of cancer cells. The release of these antigens into the tumor microenvironment aids in the activation of dendritic cells and the subsequent priming of T-cells, which are crucial for the immune response. This process is particularly beneficial when combined with immunotherapy, as the immune checkpoint inhibitors can further potentiate this immune activation by preventing the downregulation of immune responses (15). Hyperthermia increases the permeability of tumor vasculature, enhancing the delivery and penetration of chemotherapeutic agents into the tumor. This effect can be particularly significant in dense tumor tissues where drug delivery is often challenging. By improving the distribution of drugs like those in the FOLFOX regimen, hyperthermia ensures that a higher concentration of the chemotherapeutic agents reaches the tumor cells, thereby increasing their efficacy (16). While the positive outcomes in this case are encouraging, it is important to consider the generalizability of these results. Factors such as the patient’s overall health, genetic profile, and tumor characteristics can influence treatment efficacy. In our case, the patient had a specific set of conditions and previous treatments that may not be representative of all cholangiocarcinoma patients. To generalize these findings, larger studies involving diverse patient populations are necessary. Future research should aim to identify biomarkers that predict response to mEHT combined with immunotherapy and chemotherapy, ensuring that the right patients can benefit from this treatment approach. The combination of mEHT with immunotherapy and chemotherapy offers a promising approach for treating advanced cholangiocarcinoma. By enhancing immune response, improving drug delivery, and increasing tumor antigen presentation, this multimodal therapy can provide substantial benefits. However, further research is needed to validate these findings in larger, more diverse patient populations and to optimize treatment protocols for different clinical scenarios. Integrating this approach into the current treatment landscape requires careful consideration of individual patient factors and ongoing advancements in cancer therapy.

This case report highlights the significant positive outcomes achieved through the combination of immune checkpoint inhibitor (nivolumab), chemotherapy (FOLFOX), and mEHT in treating lymphatic recurrence of cholangiocarcinoma. While these results are promising, it is important to acknowledge potential selection biases inherent in case reports and to compare our findings with other published cases and studies examining similar treatment combinations to provide a balanced perspective on treatment efficacy and patient variability. In this instance, the positive outcomes—complete regression of lymph node metastasis and significant reduction in tumor marker CA 19-9 levels—might not be representative of the broader patient population with cholangiocarcinoma. Factors such as the patient’s overall health, underlying conditions, and specific tumor biology could have contributed to the favorable response observed in this case. Without a larger cohort for comparison, it is challenging to determine whether these results are widely applicable. To provide a more comprehensive view, it is essential to compare our findings with other studies that have explored similar treatment regimens. Studies such as the KEYNOTE-028 and KEYNOTE-158 trials have investigated the efficacy of pembrolizumab (an immune checkpoint inhibitor) in combination with chemotherapy for advanced biliary tract cancers, including cholangiocarcinoma. These studies have reported varying degrees of success, with some patients showing significant tumor reduction and prolonged PFS, while others did not respond as favorably (17). Sahai et al. reported on combining nivolumab with gemcitabine and cisplatin in advanced biliary tract cancers. The OS was 10.6 months, with a median PFS of 6.6 months, indicating that while some patients benefit from the combination, others may experience limited efficacy (18). Research on hyperthermia combined with radiotherapy and chemotherapy has shown enhanced treatment responses in various cancers. For example, Issels et al. demonstrated that adding regional hyperthermia to chemotherapy improved survival outcomes in patients with high-risk soft tissue sarcoma. This suggests that hyperthermia can potentiate the effects of systemic therapies, similar to the outcomes observed in our case (19). While our case report demonstrates the potential benefits of combining mEHT with immune checkpoint inhibitors and chemotherapy, it is crucial to recognize that these findings may not be universally applicable. The variability in patient responses highlights the need for personalized treatment approaches and the importance of conducting larger, randomized clinical trials to validate these results.

The management of cholangiocarcinoma, particularly in advanced stages, remains a formidable challenge due to its aggressive nature and limited treatment options. Recent advancements in cancer therapy have highlighted the potential benefits of combining various modalities to enhance treatment efficacy and improve patient outcomes. Our case report contributes to this evolving landscape by presenting a successful instance of combining immune checkpoint inhibitors, chemotherapy, and mEHT to treat lymphatic recurrence in cholangiocarcinoma. The positive outcomes observed in our case report contribute to the growing body of evidence supporting the use of multimodal therapies in cancer treatment. While our findings are based on a single case, they provide a valuable proof-of-concept that can inform future research and clinical practice. The success of the combination therapy in achieving complete regression of lymph node metastasis and significant tumor marker reduction highlights the need for further investigation through larger clinical trials.

Molecular testing, such as genomic profiling, can identify specific mutations and biomarkers that might inform targeted therapy options. These include mutations in genes like IDH1/2, FGFR2 fusions, and others with potential targeted treatments. However, in this study, we failed to provide genomic data, such as NGS results reporting the patient’s mutational profile and the implication of mutations on responses. We speculated that the availability and integration of molecular testing into routine clinical practice can vary significantly based on regional healthcare infrastructure and resources. In many regions, the cost and accessibility of comprehensive molecular profiling are limiting factors. Despite the potential benefits of targeted therapies identified through molecular testing, the multidisciplinary team, in this case, prioritized the use of accessible treatments with established efficacy.

The patient was thoroughly informed about the potential risks and benefits associated with the combined treatment regimen of immune checkpoint inhibitors, chemotherapy, and mEHT. This discussion was designed to provide a balanced view of what the patient could expect. The healthcare team explained that the combined treatment could potentially enhance therapeutic effectiveness due to the synergistic effects of mEHT with chemotherapy and immunotherapy. The patient was informed about the possibility of significant tumor shrinkage and the potential for complete regression of lymph node metastasis, as well as the overall improvement in survival and quality of life based on current research and similar case reports. The patient was also made aware of the possible side effects of chemotherapy and immunotherapy, including fatigue, nausea, and immune-related adverse events. Additionally, the risks associated with mEHT, such as skin reactions and discomfort during the procedure, were discussed. The patient was informed about the uncertainty regarding long-term outcomes and the overall effectiveness of this novel combination therapy. By involving the patient in the decision-making process and ensuring a thorough understanding of the treatment’s potential risks and benefits, the ethical transparency of this case report is enhanced. Respecting the patient’s autonomy throughout the process allowed them to make an informed decision about their treatment.

Our case report highlights the potential benefits of combining immune checkpoint inhibitors, chemotherapy, and mEHT in the treatment of advanced cholangiocarcinoma. To build on these findings and advance the field, we propose several directions for future research. The primary objective of future research should be to rigorously evaluate the efficacy and safety of the combined treatment regimen in a larger patient cohort. This can be achieved through multi-center, randomized controlled trials comparing the combination of mEHT, immunotherapy (such as nivolumab), and chemotherapy (such as FOLFOX) with standard treatments. Primary endpoints for these trials could include PFS, OS, and objective response rate (ORR), while secondary endpoints might assess quality of life, treatment tolerability, and biomarkers for treatment response. To validate the results observed in our case report across a more diverse and extensive patient population, prospective cohort studies involving patients with advanced cholangiocarcinoma receiving the combination therapy should be conducted. These studies should collect detailed data on patient demographics, treatment regimens, outcomes, and adverse events. Comparative effectiveness analysis can then be performed to evaluate the real-world impact of the combined treatment on survival and disease progression, with subgroup analyses identifying patient characteristics associated with better outcomes. Preclinical studies are essential to understand the underlying mechanisms by which mEHT enhances the effects of immunotherapy and chemotherapy. Laboratory studies using cell lines and animal models of cholangiocarcinoma should investigate the biological processes involved. Key areas of investigation should include the role of heat shock proteins in tumor immunogenicity and response to immune checkpoint inhibitors, the impact of hyperthermia on tumor vasculature and drug delivery efficiency, and the interactions between mEHT-induced stress responses and immune activation. Identifying predictive biomarkers that can help select patients most likely to benefit from the combined treatment regimen is crucial. Correlative studies within clinical trials and observational studies should collect tissue and blood samples for molecular analysis. Using genomics, proteomics, and immunohistochemistry, researchers can identify biomarkers associated with response to mEHT, immunotherapy, and chemotherapy. Potential biomarkers could include specific genetic mutations, protein expression levels, and immune cell infiltration patterns. Future research should focus on validating and expanding upon the promising results observed in this case report. By conducting rigorous clinical trials, larger observational studies, and detailed preclinical investigations, we can better understand the interactions between these therapies and optimize treatment protocols. Identifying predictive biomarkers will further enable personalized treatment approaches, ultimately improving outcomes for patients with advanced cholangiocarcinoma. These efforts will contribute significantly to the advancement of cholangiocarcinoma treatment and potentially benefit other cancer types as well.

## Conclusion

The effective treatment of lymphatic recurrence in cholangiocarcinoma by combining immunotherapy, chemotherapy, and modulated electro-hyperthermia emphasizes the significance of innovative and multidisciplinary approaches in complex cancer cases. More research and clinical studies are needed to explore the potential of such multiple strategies in enhancing outcomes for patients with advanced cholangiocarcinoma.

## Data Availability

The original contributions presented in the study are included in the article/supplementary material. Further inquiries can be directed to the corresponding author.
